# Twenty-four-hour time-use composition and cognitive function in older adults: cross-sectional findings of the ACTIVate study

**DOI:** 10.3389/fnhum.2022.1051793

**Published:** 2022-11-24

**Authors:** Maddison L. Mellow, Dorothea Dumuid, Alexandra T. Wade, Ty Stanford, Timothy S. Olds, Frini Karayanidis, Montana Hunter, Hannah A. D. Keage, Jillian Dorrian, Mitchell R. Goldsworthy, Ashleigh E. Smith

**Affiliations:** ^1^Alliance for Research in Exercise, Nutrition and Activity, Allied Health and Human Performance, University of South Australia, Adelaide, SA, Australia; ^2^Functional Neuroimaging Laboratory, School of Psychological Sciences, The University of Newcastle, Newcastle, NSW, Australia; ^3^Healthy Minds Research Program, Hunter Medical Research Institute (HMRI), Newcastle, NSW, Australia; ^4^Behaviour-Brain-Body Research Centre, Justice and Society, University of South Australia, Adelaide, SA, Australia; ^5^Lifespan Human Neurophysiology Group, School of Biomedicine, University of Adelaide, Adelaide, SA, Australia; ^6^Hopwood Centre for Neurobiology, Lifelong Health Theme, South Australian Health and Medical Research Institute (SAHMRI), Adelaide, SA, Australia

**Keywords:** time use, cognitive function, ageing, sleep, sedentary behaviour, physical activity

## Abstract

**Introduction:**

Physical activity, sedentary behaviour and sleep are associated with cognitive function in older adults. However, these behaviours are not independent, but instead make up exclusive and exhaustive components of the 24-h day. Few studies have investigated associations between 24-h time-use composition and cognitive function in older adults. Of these, none have considered how the quality of sleep, or the context of physical activity and sedentary behaviour may impact these relationships. This study aims to understand how 24-h time-use composition is associated with cognitive function across a range of domains in healthy older adults, and whether the level of recreational physical activity, amount of television (TV) watching, or the quality of sleep impact these potential associations.

**Methods:**

384 healthy older adults (age 65.5 ± 3.0 years, 68% female, 63% non-smokers, mean education = 16.5 ± 3.2 years) participated in this study across two Australian sites (Adelaide, *n* = 207; Newcastle, *n* = 177). Twenty-four-hour time-use composition was captured using triaxial accelerometry, measured continuously across 7 days. Total time spent watching TV per day was used to capture the context of sedentary behaviours, whilst total time spent in recreational physical activity was used to capture the context of physical activity (i.e., recreational accumulation of physical activity vs. other contexts). Sleep quality was measured using a single item extracted from the Pittsburgh Sleep Quality Index. Cognitive function was measured using a global cognition index (Addenbrooke’s Cognitive Examination III) and four cognitive domain composite scores (derived from five tests of the Cambridge Neuropsychological Test Automated Battery: Paired Associates Learning; One Touch Stockings of Cambridge; Multitasking; Reaction Time; Verbal Recognition Memory). Pairwise correlations were used to describe independent relationships between time use variables and cognitive outcomes. Then, compositional data analysis regression methods were used to quantify associations between cognition and 24-h time-use composition.

**Results:**

After adjusting for covariates and false discovery rate there were no significant associations between time-use composition and global cognition, long-term memory, short-term memory, executive function, or processing speed outcomes, and no significant interactions between TV watching time, recreational physical activity engagement or sleep quality and time-use composition for any cognitive outcomes.

**Discussion:**

The findings highlight the importance of considering all activities across the 24-h day against cognitive function in older adults. Future studies should consider investigating these relationships longitudinally to uncover temporal effects.

## Introduction

The positive relationship between physical activity and cognitive function in older adulthood is well documented. Several cross-sectional studies have demonstrated that older adults who engage in higher levels of physical activity have better global cognitive function (Falck et al., 2017b), executive function (Daly et al., 2015), and memory (Xu et al., 2011). Further, some longitudinal (Daly et al., 2015; Rojer et al., 2021) and intervention studies (Lautenschlager et al., 2008; Northey et al., 2018) suggest that physical activity engagement is associated with lower odds of cognitive decline and preserved cognitive functioning in older adults. Conversely, other studies have found no associations between physical activity and cognitive function in older adults after adjusting for sociodemographic and health covariates such as age, sex, education, smoking, body mass index and depression, all of which are risk factors for cognitive decline and dementia (Daimiel et al., 2020; Livingston et al., 2020).

Several studies have reported intensity-specific differences in the association between physical activity and cognitive function. Moderate-to-vigorous physical activity (MVPA) is strongly and positively associated with cognitive function (Kerr et al., 2013; Rojer et al., 2021), and associations between engagement in light-intensity physical activity (LPA) and cognitive function have also been reported (Johnson et al., 2016). Additionally, there is evidence to suggest that certain types of physical activity may be more beneficial for cognitive function than others because they differ in level of cognitive engagement. Physical activity may be accumulated in a variety of contexts, including occupational, household, transportation, and recreational modalities (Gill et al., 2015). Activities which constitute recreational physical activity (also known as leisure-time physical activity), such as dancing, gym classes or playing sports, are considered to be more beneficial for cognitive function than other physical activity modalities such as active transport (i.e., walking to the bus stop) or household chores (Phansikar et al., 2019), whilst studies on the impact of occupational physical activity on cognitive function in older adults have presented mixed findings (Rovio et al., 2007; Adam et al., 2013). The benefits associated with recreational physical activity may be partly because many recreational activities require more neuromuscular complexity, higher levels of cognitive engagement, and more social interaction (i.e., through engaging with others during group activities) (Phansikar et al., 2019). However, to date, few studies have assessed differences between recreational physical activity and non-recreational physical activity against cognitive function in older adults, and this warrants further exploration.

A limitation of many studies in this field is that they have not considered the interaction between physical activity and other time-use behaviours, sedentary behaviour and sleep, which make up the 24-h day (Mellow et al., 2022). There is mixed evidence regarding the association between sedentary behaviour and cognitive functioning in older adults. Engaging in excessive daily sedentary behaviour has been negatively associated with cognitive function in older adults in a previous review and longitudinal study (Falck et al., 2017a; Ku et al., 2017). Conversely, both Maasakkers et al. (2020) and Chen et al. (2022) did not find associations between *total* sedentary time and cognitive function in older adults, although Chen et al. (2022) did report a negative association between prolonged *bouts* of sedentary behaviour and cognitive outcomes. These discrepant findings may reflect the importance of considering how sedentary behaviour is broken up, or types of sedentary behaviour, rather than operationalizing sedentary behaviour as total undifferentiated sedentary time for cognitive function outcomes (Maasakkers et al., 2020). The context of sedentary behaviour may differentially affect cognitive functioning, such that sedentary behaviours that are more cognitively engaging (such as computer use or work-related activities) may have different associations with cognitive function outcomes compared to cognitively passive sedentary behaviours [such as television (TV) watching]. For example, several studies have reported positive associations between computer use and cognitive outcomes, and negative associations between TV watching and cognitive outcomes (Kesse-Guyot et al., 2012; Bakrania et al., 2018). Olanrewaju et al. (2020) reported no cross-sectional associations between total sitting time and cognitive function but found significant negative associations between TV watching and verbal memory and fluency. Taken together, when investigating cognitive function outcomes, it may be useful to consider the context of sedentary behaviours (i.e., how mentally engaging they are), in addition to total sedentary time.

Sleep is also associated with cognitive function in older adults. Several studies have suggested that too much or too little sleep (i.e., >9 h or <6 h) is negatively associated with cognitive function, often referred to as an “inverted U-shaped” relationship (Gildner et al., 2014; Wild et al., 2018). Other studies found no evidence for these relationships (McSorley et al., 2019) or reported associations with long but not short sleep duration (Faubel et al., 2009; Low et al., 2019; Kondo et al., 2021). Beyond sleep duration, poor sleep quality and efficiency have been linked to poor cognitive function in older adults (Nebes et al., 2009; Miyata et al., 2013). Therefore, in the context of cognitive function in ageing, it is likely important to consider the not only sleep duration, but the whole sleep experience including sleep quality.

It is plausible that both the amount of time and the characteristics of that time use are important factors that may affect cognitive function. Furthermore, physical activity, sleep and sedentary behaviour interact to make up the 24-h day, such that increasing time in one behaviour must result in an equal and opposite change in time spent in one or both of the other time-use behaviours (Dumuid et al., 2020). Intervention studies in which participants only modify one daily activity (e.g., increase physical activity) are not able to disentangle whether any cognitive or other benefits are due to the change in this activity (e.g., the increase in physical activity) or to the compensatory change in time spent in one of the other behaviours (i.e., decreased sedentary behaviour or sleep) (Dumuid et al., 2017; Rosenberger et al., 2019). Recent developments in statistical techniques based on compositional data analysis (CoDA) (Aitchison, 1982) allow the daily composition of all three time-use behaviours, or *time-use composition*, to be studied against health outcomes in a single analytical model. CoDA requires time-use variables (e.g., physical activity, sedentary behaviour, and sleep) to be expressed as isometric log ratios that are able to be used in traditional statistical models, such as multiple linear regression analyses. A CoDA approach overcomes the issue of the perfect multicollinearity of 24-h time use data that violates the assumptions of many statistical models. This has previously been (inadequately) overcome by omitting one or more time-use behaviours from statistical models (Dumuid et al., 2017).

To our knowledge, only three previous studies on time use and cognitive function in older adults have considered all three time-use behaviours simultaneously in the same statistical model. Briefly, using isotemporal substitution methods, Fanning et al. (2017) reported that replacing time in sedentary behaviour with MVPA *or* sleep was positively associated with cognitive function, whilst replacing time in sedentary behaviour with LPA had no association with cognitive function. Wei et al. (2021) reported that the associations between time-use composition and cognitive function varied between older adults who achieved <7 h of sleep per night compared to those who achieved >7 h per night. However, these studies relied on a combination of device-based and self-report measures to capture 24-h time use, which may have resulted in the over- or under-estimation of time spent in respective behaviours. Only one study has captured 24-h time use using device-based measures and investigated these relationships using a CoDA approach. Dumuid et al. (2022) reported that 24-h time-use composition was associated with global cognition and executive function in a sample of middle-to-older adults. Predictive modelling indicated that more time in MVPA and less time in LPA was beneficial for cognitive function, and that this relationship was more pronounced in people at higher genetic risk of Alzheimer’s disease (carrying an APOE ε4 allele) (Dumuid et al., 2022). Together, these studies highlight the importance of simultaneously considering all behaviours across the 24-h day against cognitive function. However, none have considered the context of time-use behaviours in addition to their duration (e.g., sleep quality and context of physical activity and sedentary behaviour).

This study investigates the associations between 24-h time-use composition of physical activity, sedentary behaviour, and sleep on cognitive function in a large sample of healthy older Australians. It uses data collected during the baseline phase of the ACTIVate study, a 3-year multisite study aiming to optimise daily activity patterns and diet for dementia prevention [see Smith et al. (2022) for study protocol]. In addition to examining the relative duration of these behaviours across the 24-h cycle, we investigate whether the context of physical activity, sedentary behaviour and quality of sleep modify the associations between time-use composition and cognitive outcomes.

## Materials and methods

### Ethics

The ACTIVate study was registered with the Australian New Zealand Clinical Trials Registry (ACTRN12619001659190) on November 27, 2019. Ethics approval was obtained from the University of South Australia and University of Newcastle Human Research Ethics Committee (202639). All procedures were conducted in accordance with the Declaration of Helsinki.

### Participant recruitment and screening

Eligibility criteria for the ACTIVate study are described in more detail elsewhere (Smith et al., 2022). Briefly, participants met the inclusion criteria if they were aged 60–70 years, fluent in English, had no current clinical diagnosis of dementia, major neurological or psychiatric diagnoses, known intellectual disability, or major physical disability, and presented no contraindications to transcranial magnetic stimulation screening (Rossi et al., 2009). Potential participants were required to undertake a phone screening interview, during which the inclusion criteria above were assessed (via self-report) in additional to completing a Montreal Cognitive Assessment (Blind) (MoCA-B) to screen for dementia (using a cut-off score of <13/22).

Power calculations were determined for the larger ACTIVate study based on cross-sectional pilot data, in the absence of longitudinal data on diet and activity compositions in relation to cognitive outcomes. Aiming for 80% power, accounting for the longitudinal design and allowing for attrition and response rate at recruitment, the final sample size of 448 participants was determined [see Smith et al. (2022) for further details].

### Study measures

#### Device-measured activity patterns

Time spent in physical activity, sedentary behaviour and sleep was measured using accelerometry. Participants were asked to wear a triaxial accelerometer on their non-dominant wrist (Axivity AX3) 24 h per day for seven consecutive days, with data recorded at a sampling frequency of 100 Hz. Raw acceleration data were downloaded using the Open Movement GUI software (OmGUI; Newcastle, UK) and further processed using a custom MATLAB graphic user interface developed at the University of South Australia (COBRA; MATLAB R2018B). Time spent in sleep was verified by manual cross-checking of sleep logs completed by participants during the 7 days of recording and visual inspection of the accelerometry trace across the 7 days. Non-wear time was identified as ≥60 min of ≤25 g-min, and was also manually verified against participant diaries (removal of watch recorded as well as sleep information) and by visual inspection of the accelerometry trace. Waking day behaviours were classified as time in MVPA (>93 g-min), LPA (>48 g-min) or sedentary behaviour (<48 g-min) using previously published cut points adjusted for sampling frequency (Hildebrand et al., 2014).

Accelerometry data were classified as a “valid wear day” if the accelerometer was worn for at least 10 waking hours. Only participants with three or more valid weekdays and one valid weekend day were included in the analyses. Total time spent in each activity was averaged across the recording period, providing average time (minutes) spent in MVPA, LPA, sedentary behaviour, and sleep per day.

#### Self-report activity measures

##### Television watching

The amount of TV watching per day (in minutes) was used as a measure for cognitive engagement during sedentary behaviour. These data were obtained through the Multimedia Activity Recall for Children and Adults (MARCA), a computerised use-of-time recall tool containing over 500 potential daily activities (Ridley et al., 2006). During the MARCA assessment, participants were asked to recall every activity they had engaged in over the 2 previous days (Gomersall et al., 2011). Due to study constraints, not all participants recalled one weekend day and one weekday (two weekdays most commonly recalled). All MARCA phone calls were scheduled during the 7-days that participants were wearing the accelerometer. Total time spent watching TV was averaged across the 2 days of recall and used to categorise participants into low, medium, and high tertiles.

##### Recreational physical activity

To investigate whether the context of physical activity influenced associations between time-use composition and cognitive function, we captured total time spent in recreational physical activity using the MARCA assessments (Ridley et al., 2006). Recreational physical activity encompasses play-based activities (e.g., totem tennis, darts, juggling) and sport (e.g., dancing, gym-based exercise, team sports, partner sports and individual sports). Total time spent in recreational physical activity was averaged across the 2 days of recall, and participants were further categorised in to “no recreational physical activity,” “0–30 min of recreational physical activity,” and “30+ minutes of recreational physical activity” categories. These categories were chosen during analysis because (1) there were a considerable number of participants with 0 min of recreational physical activity, and (2) separating those who did engage in recreational physical activity in to <30 min or >30 min allows for sufficient comparison (rather than comparing “none” against “some” recreational physical activity). An exhaustive list of activities that were categorised as recreational physical activity can be found in Supplementary material 1.

##### Sleep quality

The Pittsburgh Sleep Quality Index (PSQI) is a subjective measure of sleep quality over the past month (Buysse et al., 1989). The PSQI asks 19 questions which produce seven component scores assessing sleep quality, latency, duration, efficiency, disturbances, use of medications and daytime dysfunction. For the purpose of this study, the sleep quality component was used as a measure of sleep quality (question 6: “*During the past month, how would you rate your sleep quality overall?”*). Participants rated their sleep using the following scoring system: 0 = “very good”; 1 = “fairly good”; 2 = “fairly bad”; 3 = “very bad.” Participants were therefore categorised as having “bad” sleep quality (scoring 2 or 3) or “good” sleep quality (scoring 0 or 1) for final analyses.

#### Cognitive function measures

##### Addenbrooke’s Cognitive Examination III

The Addenbrooke’s Cognitive Examination III (ACE-III) was used as a measure of global cognition. The ACE-III is a brief paper-and-pencil-style cognitive screening tool that assesses cognitive function across five domains as a total score out of 100 that includes scoring of memory, attention/orientation, language, fluency and visuospatial ability. The ACE-III demonstrates high internal reliability (Cronbach’s α = 0.88), and high specificity (0.96) and sensitivity (1.00) to detecting dementia using the cut-off score of 88/100 (Hsieh et al., 2013).

##### Cambridge Automated Neuropsychological Test Automated Battery

Domain-specific cognitive function was assessed using the Cambridge Automated Neuropsychological Test Automated Battery (CANTAB). CANTAB is a computerised neuropsychological test battery containing a range of cognitive tests across several cognitive domains. CANTAB tests have demonstrated discriminant validity between clinical populations and healthy controls (Saunders and Summers, 2010), and moderate correlations with traditional neuropsychological tests in younger populations (e.g., Trail Making Test with Paired Associates Learning; Animal Fluency and Green Story Delayed Recall with Verbal Recognition Memory) (Smith et al., 2013) and older populations (e.g., Rey complex figure test compared to Paired Associates Learning test) (Lenehan et al., 2015). Test-retest reliability of CANTAB tests ranges from weak to strong (0.56 to.89) (Gonçalves et al., 2016).

Participants completed the following tests: Paired Associates Learning (PAL), Reaction Time (RTI), Multitasking Test (MTT), Verbal Recognition Memory (immediate and delayed) (VRM), and One Touch Stockings of Cambridge (OTS). The total time to complete all five CANTAB tasks including a 2-min familiarisation task (Motor Screening Task) was approximately 40 min.

For tests where lower scores indicate better performance (e.g., reaction time), scores were reversed. Raw CANTAB test scores were then converted to *z*-scores. Individual outcome measure *z*-scores were collated into cognitive composites using the Cattell-Horn-Carroll-Miyake (CHCM) cognitive domain taxonomy as a guiding framework (Webb et al., 2018). Cognitive composites were classified according to the broad domains in the CHCM taxonomy: long-term storage and retrieval (herein referred to as “long-term memory”; short-term and working memory (herein referred to as “short-term memory”); executive function; and processing speed. For tests that were not classified within the CHCM taxonomy (i.e., OTS), tests were allocated to a cognitive domain by consensus among the authorship team. Table 1 displays the outcome measures used to create each cognitive composite.

**TABLE 1:** Cambridge Automated Neuropsychological Test Automated Battery outcome measures and composite scores.

Cognitive domain	Cognitive test	Outcome measures (score range)
Global cognition	Addenbrooke’s Cognitive Examination III	Total score (0–100)
Long-term memory	Verbal Recognition Memory	Delayed recognition total correct (0–36)
Short-term memory	Verbal Recognition Memory	Immediate recognition total correct (0–36)
		Immediate free recall total correct distinct words (0–18)
	Paired Associates Learning	Total errors (adjusted) (0–70)*
		First attempt memory score (0–20)
Executive function	Multitasking test	Total incorrect responses (0–160)*
		Median response latency multitasking cost (–1,900 to 1,900)*
		Median response latency incongruency cost (–1,900 to 1,900)*
	One Touch Stockings of Cambridge	Problems solved on first choice (0–15)
		Median latency to first choice (0 to ∞)*
Processing speed	Reaction time	Median simple reaction time (100–5,100)*
		Median 5-choice reaction time (100–5,100)*
		Median simple movement time (100–5,100)*
		Median 5-choice movement time (100–5,100)*

*Denotes scores that were reversed (so that higher scores = better performance).

For each outcome measure to be included in the cognitive composite, a selection of inclusion criteria was applied to respective CANTAB tests. Participants who achieved <50% accuracy on tests used in the criteria listed in Supplementary material 2 were excluded from analyses. Cognitive composite scores were not generated for a participant unless all data were available (i.e., four test scores in a four-test composite), and participants met all inclusion criteria within that composite.

#### Covariates

Demographic factors that have been associated with increased dementia risk (Livingston et al., 2020) were entered as covariates, including age (years), sex (male, female), education (total years), and smoking status (current smoker, previous smoker, never smoker). Additionally, models were adjusted for site (Adelaide, Newcastle).

### Statistical analysis

#### Associations between time-use composition and cognitive function

All inferential statistics were conducted in R version 4.2 (R Core Team, 2021), and the code used to analyze data is available at https://github.com/MaddisonMellow/Time-use-cognition-paper. Pearson correlation coefficients between time use variables (MVPA, LPA, sedentary behaviour and sleep) and cognitive outcomes (global cognition, short-term memory, long-term memory, executive function and processing speed) were initially calculated pairwise to assess univariate associations. Next, compositional analyses were conducted using the *compositions* package (Van den Boogaart and Tolosana-Delgado, 2008). Daily time-use compositions were created for each participant, representing the average proportion of time spent in MVPA, LPA, sedentary behaviour and sleep each day (distinct and exhaustive categories summing to 1,440 min of the day).

In order to include time-use composition as a variable in linear regression models, the composition of behaviours were expressed as isometric log ratio coordinates (see Dumuid et al. (2017) for extensive overview of this approach). To achieve this for a four-part composition, three isometric log ratios were created: the first represented the log-ratio of one behaviour (e.g., sleep) to the remaining three behaviours (e.g., sedentary behaviour, LPA, MVPA); for the second isometric log-ratio coordinate, sleep was excluded and the log-ratio of the next time-use behaviour in the set (sedentary behaviour) to the remaining two behaviours (LPA and MVPA) was calculated; finally, the third isometric log-ratio coordinate only contained information on the remaining two behaviours (LPA:MVPA). All three isometric log-ratio coordinates were entered into the regression model to represent the entire 24-h time-use composition. Further, all three coordinates were included in ANOVA type II F-tests (described below) to test the null hypothesis that all coefficients of isometric log-ratios were equal to zero. With the aim of producing parsimonious models supported by the data for each outcome variable, backward selection of the multiple linear regression models was employed. The initial (potential) model for each outcome included an intercept, main effects (demographics, time-use composition, TV watching, recreational physical activity level, sleep quality) and the two-way interaction effects of primary interest in time-use composition with each TV watching, recreational physical activity, and sleep quality. The backward selection process proceeded by simplifying the model by sequential *F*-tests, stopping model reduction when significance at the level of 0.05 was reached for all remaining terms in the following groups, considering the following order: (i) interaction terms (in order of largest p-value), then (ii) the time-use composition, and (iii) the remaining covariates collectively. Type II *F*-tests were used to determine variable significance, which assesses variable effects after adjusting for other variables while adhering to the principle of marginality (Langsrud, 2003; Fox and Weisberg, 2019). *P*-values within each of the final models were adjusted for multiple comparisons using the Benjamini-Hochberg false discovery rate adjustments (Benjamini and Hochberg, 1995). Both adjusted and unadjusted *p*-values are presented in the results.

#### Interaction terms and reallocations of time

Where models containing interaction terms remained statistically significant after false discovery rate adjustment, we planned to plot model-generated one-for-remaining predictive response curves to show how the cognitive outcome measures were associated with meaningful reallocations of time (i.e., in 15-min increments), across different levels of sleep quality, TV watching or recreational PA [see Dumuid et al. (2017) for example].

## Results

### Participant demographics

Four hundred and twenty-six participants were initially included in the dataset. Of these, 21 were removed as they did not have valid accelerometry data, and 21 were removed due to missing covariate data. Thus, the overall final sample included 384 older adults (65.5 ± 3.0 years old, 121 males). Means and standard deviations of demographic and other key variables are presented in Table 2. The final sample were 68% female, 63% non-smokers, with mean 16.5 ± 3.2 years of total education. Over half of participants (*n* = 207) reported engaging in no recreational physical activity, whilst 26.7% engaged in over 30 min per day.

**TABLE 2:** Participant demographics.

Variable	Level	Adelaide (*n* = 207)	Newcastle (*n* = 177)	Total (*n* = 384)
Age		65.6 ± 2.8	65.4 ± 3.2	65.5 ± 3.0
Sex	Female	165	98	263
	Male	42	79	121
Education (years)		16.3 ± 3.3	16.7 ± 3.2	16.5 ± 3.2
Smoking status	Current smoker	84 (41%)	59 (33%)	143 (37%)
	Previous smoker	7 (3%)	0 (0%)	7 (2%)
	Never smoked	116 (56%)	118 (67%)	234 (61%)
Device-measured PA levels (min/day)	MVPA	91 ± 46	86 ± 47	89 ± 47
	LPA	178 ± 48	178 ± 52	178 ± 50
	SB	657 ± 90	682 ± 87	668 ± 90
	Sleep	513 ± 59	492 ± 53	503 ± 57
Sleep quality rating	Good	165 (79.7%)	149 (84.2%)	314 (81.6%)
	Bad	42 (20.3%)	28 (15.8%)	70 (18.8%)
Recreational PA (min/day)	“None”	0	0	0
	“<30”	21 ± 8	17 ± 8	19 ± 8
	“>30”	80 ± 49	81 ± 59	80 ± 53
TV watching (min/day)	High	223 ± 48	225 ± 67	224 ± 59
	Medium	128 ± 21	123 ± 17	126 ± 20
	Low	43 ± 28	47 ± 33	44 ± 30
ACE-III score		95.8 ± 3.1	94.2 ± 3.9	95.1 ± 3.6

Values are presented as either mean ± SD for numeric variables or count (percentage) for categorical variables. Recreational physical activity (PA) and TV watching data are presented as the mean ± SD minutes per day spent in respective activities. MVPA, moderate-vigorous physical activity; LPA, light physical activity; SB, sedentary behaviour; PME, perceived mental effort; ACE-III, Addenbrooke’s Cognitive Examination III.

**FIGURE 1:**
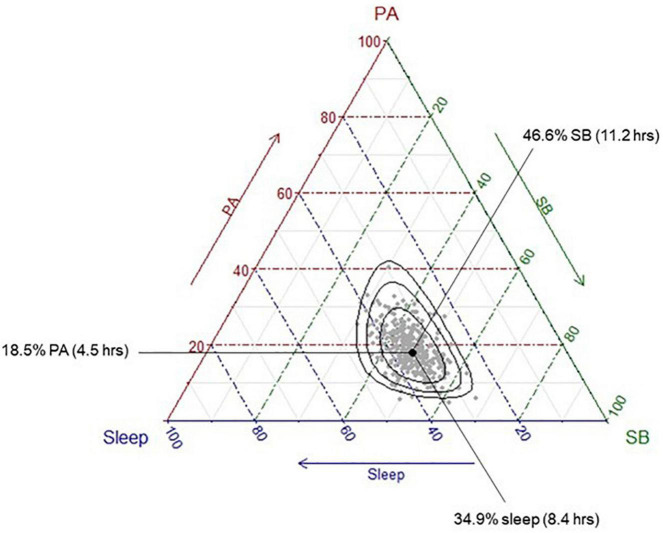
Distribution of the sample time-use compositions. Each grey dot represents a single participant’s time-use composition. The black dot represents the average time-use composition of the sample, calculated as the geometric means of each activity, collectively adjusted to sum to 1,440 min. This differs to the arithmetic means, which are presented in Table 2; instead of summing the values and dividing by the number of values “*n*,” the geometric mean multiplies all the values and then takes the *n*th root. To convert the geometric mean to the compositional mean, the “closure” operator (a function part of Compositions package in R) is applied to the geometric mean, so that the centrality measure reflects the 1,440 min (24 h) in the day. The black dot represents this average composition (“compositional mean”) and shows that the average participant spends approximately 18.5% of their day in physical activity, 46.6% of their day in sedentary behaviour, and 35% of their day in sleep. Black ellipses represent 75, 95, and 99% confidence intervals, respectively.

A number of participants were removed from each analysis due to missing cognitive data: total samples for each cognitive outcome included: *n* = 384 for global cognition; *n* = 292 for short-term memory; *n* = 353 for long-term memory; *n* = 369 for executive functions; *n* = 358 for processing speed. Participants’ time-use compositions (i.e., the average proportion of the day spent in sleep, sedentary behaviour and physical activity across all participants) are presented in Figure 1. The ternary diagram (3-simplex, a triangle) contains all possible combinations of three time-use categories in a day. The values of the compositional parts of a point on the simplex can be observed as the proportional distance of the point toward a compositional part’s vertex, perpendicular to the opposing side’s line. For example, the compositional mean is 34.9% of the distance toward the sleep-labelled vertex perpendicular from the side between the physical activity and sedentary behaviour labels. Similarly, the values for physical activity and sedentary behaviour of 18.5 and 46.6%, respectively, can be observed using the same method. On average, participants spent approximately 4.5 h per day in physical activity (across both moderate-vigorous and light intensities), 8.4 h in sleep, and 11.2 h in sedentary behaviour).

### Associations between time-use composition and cognition

#### Pairwise correlations

Pearson correlation coefficients revealed that time spent in sleep was negatively correlated with long term memory (*r* = –0.11, *p* = 0.03), time spent in sedentary behaviour was negatively correlated with processing speed (*r* = –0.13, *p* = 0.01), and time spent in MVPA was positively correlated with processing speed (*r* = 0.17, *p* < 0.01) (Table 3).

**TABLE 3:** Pairwise correlations between time-use variables and cognitive outcomes.

	ACE-III	Long-term memory	Short-term memory	Executive function	Processing speed	Sleep (min)	SB (min)	LPA (min)
Long-term memory	0.22**							
Short-term memory	0.31**	0.57**						
Executive function	0.16**	0.22**	0.21**					
Processing speed	0.09	0.06	0.03	0.03				
Sleep (min)	0.00	−−0.11*	–0.06	0.02	0.00			
SB (min)	0.00	0.05	0.05	0.01	−−0.13*	−−0.45**		
LPA (min)	–0.04	0.02	–0.01	–0.05	0.06	−−0.16**	−−0.68**	
MVPA (min)	0.03	0.04	0.01	0.01	0.17**	−−0.14**	−−0.65**	0.43**

Data are presented as Pearson correlation coefficients (r). Bold denotes that the p-value is statistically significant. *Denotes p-values ≤ 0.05. **Denotes p-values ≤ 0.01. ACE-III, Addenbrooke’s Cognitive Examination III; SB, sedentary behaviour; LPA, light physical activity; MVPA, moderate to vigorous physical activity.

#### Linear regression models

Prior to adjustment for false discovery rate, time-use composition was significantly associated with processing speed, such that more time spent in sleep (β = 0.28) or MVPA (β = 0.20) relative to time spent in the remaining behaviours was associated with faster processing speed, whilst more time spent in LPA (β = –0.16) or sedentary behaviour (β = –0.33) was associated with slower processing speed (Table 4). Additionally, several covariates were significantly associated with cognitive outcomes: older age was associated with poorer executive function (β = –0.05) and slower processing speed (β = –0.04); site was negatively associated with global cognition (β = –1.43) and positively associated with long-term memory (β = 0.25) and short-term memory (β = 0.17) (i.e., participants from Newcastle had lower global cognition scores and higher long-term and short-term memory scores than Adelaide); higher education (years) was associated with better global cognition (β = 0.22); sex (being female) was negatively associated with executive function (β = –0.23) and engaging in no recreational physical activity (relative to >30 min) was associated with poorer long-term memory (β = –0.38). None of the interaction terms were statistically significant.

**TABLE 4:** Statistical output of ANOVA type II F-tests for cognitive outcomes.

	Global cognition			Long-term memory			Short-term memory			Executive function			Processing speed		
	*F*_(n,d)_	*p*-value	*adj.p*	*F*_(n,d)_	*p*-value	*adj.p*	*F*_(n,d)_	*p*-value	*adj.p*	*F*_(n,d)_	*p*-value	*adj.p*	*F*_(n,d)_	*p*-value	*adj.p*
Age	0.68_(1, 372)_	0.41	0.54	1.18_(1, 354)_	0.28	0.32	2.52_(1, 293)_	0.11	0.31	29.16_(1, 357)_	**<0.01**	**<0**.01*	7.74_(1, 359)_	**≤0**.**01**	0.05
Sex	1.20_(1, 372)_	0.27	0.44	3.98_(1, 354)_	0.05	0.13	0.05_(1,293)_	0.81	0.81	15.73_(1, 357)_	**<0**.**01**	**<0**.01*	0.17_(1, 359)_	0.68	0.96
Site	15.20_(1, 372)_	**≤0**.**01**	**≤0**.01*	5.19_(1, 354)_	**0**.**02**	0.09	6.14_(1,293)_	**0**.**01**	0.11	0.70_(1, 357)_	0.40	0.49	3.05_(1, 359)_	0.08	0.24
Smoking status	0.75_(2,372)_	0.47	0.54	2.28_(2,354)_	0.10	0.17	2.08_(2,293)_	0.13	0.31	0.68_(2,357)_	0.51	0.51	0.08_(2,359)_	0.92	0.96
Education (years)	16.44_(1, 372)_	**≤0**.**01**	**≤0**.01*	2.13_(1, 354)_	0.15	0.19	1.13_(1,293)_	0.29	0.45	3.81_(1, 357)_	0.05	0.10	0.10_(1, 359)_	0.75	0.96
Sleep quality	3.43_(3,372)_	0.06	0.13	0.04_(3,354)_	0.85	0.85	0.62_(3,293)_	0.43	0.49	0.64_(3,357)_	0.43	0.49	0.30_(3,359)_	0.58	0.96
TV time (min/day)	2.78_(2,372)_	0.06	0.13	2.29_(2,354)_	0.10	0.16	1.88_(2,293)_	0.15	0.31	0.97_(2,357)_	0.38	0.49	0.03_(2,359)_	0.97	0.97
Recreational PA (min/day)	0.36_(2,372)_	0.70	0.70	5.15_(2,354)_	**≤0**.**01**	**0**.05*	1.09_(2,293)_	0.34	0.45	3.09_(2,357)_	**0**.**05**	0.10	1.33_(2,359)_	0.27	0.60
Time-use composition	–	–	–	–	–	–	–	–	–	–	–	–	2.87_(3,359)_	**0**.**04**	0.16

F_(n_,_d)_, F statistic, and numerator and denominator degrees of freedom; adj.p, p-value adjusted for false discovery rate. Bold denotes statistical significance (p ≤ 0.05). *Denotes p-values that remained significant after false discovery rate adjustment. “–” Denotes variables that were not included in final models for respective cognitive outcomes. Interaction terms (for sleep quality, recreational PA or TV watching) were not included in final models for any cognitive outcomes and therefore are not listed in this table.

After false discovery rate adjustment, none of the associations between 24-h time-use composition and cognitive function outcomes were statistically significant. Associations between age and executive functions, recreational physical activity and long-term memory, sex and executive functions, as well as education, site and global cognition, remained significant. Unadjusted and adjusted *p*-values for all variables across each cognitive outcome are displayed in Table 4. Linear regression outputs for each cognitive outcome can be found in Supplementary material 3.

## Discussion

### 24-h time use composition and cognitive function in older adults

Although there is some evidence that physical activity, sleep, and sedentary behaviour are independently associated with cognitive function in older adults, it remains unclear how the balance of these three behaviours in the 24-h day, or time-use composition, relates to cognitive function outcomes. The current study investigated the cross-sectional associations between 24-h time-use composition and cognitive function in a sample of healthy older adults. We initially explored pairwise correlations to understand the independent and unadjusted associations between time use variables and cognitive outcomes, in which we found that sleep was negatively correlated with long-term memory, sedentary behaviour was negatively correlated with processing speed, and MVPA was positively correlated with processing speed. Subsequently, after adjusting for demographic and health factors that are associated with risk of dementia (age, sex, education, smoking status), linear regression models demonstrated 24-h time-use composition was significantly associated with processing speed, but there were no associations with global cognition, long-term memory, short-term memory or executive function. However, the association between 24-h time use and processing speed was non-significant after adjustment for false discovery rate. Together, these findings demonstrate the importance of considering all activities across the 24-h day against cognitive function, as the relationships between time-use behaviours and several cognitive outcomes (assessed *via* correlations) were likely attenuated after accounting for other time-use behaviours.

Few studies have examined 24-h day time use against cognitive function in older adults, and only one of these has used CoDA (Dumuid et al., 2022). Our findings of no associations contradict the few previous studies that reported associations between time-use composition and cognitive function in healthy older adults (Fanning et al., 2017; Wei et al., 2021). There are several important differences which may explain the contradictory findings in the current study, such as the type of analyses conducted (testing associations of overall compositions rather than time-reallocations with cognitive function), and the characteristics of the recruited samples. Both Fanning et al. (2017) and Wei et al. (2021) recruited a low-active sample, whereby participants engaged in approximately 46 min or 36 min of MVPA per day, respectively. In our sample, participants were achieving an average of 89 min/day in MVPA. As such, in a low-active sample, it is likely that small differences in MVPA (or reallocations of time to MVPA) have more potent effects on cognitive function as the level of MVPA is low across participants. For example, in participants who engage in lower levels of MVPA per day (i.e., 30 min/day), reallocating an additional 15 min of MVPA per day equates to a 50% increase in total MVPA, whereas for those with a baseline level of 90 min of MVPA per day, reallocating an additional 15 min is equivalent to a ∼16% increase only. Additionally, Dumuid et al. (2022) recruited a sample from a wider age range (50–80 years), across a range of cardiovascular risk profiles (“low” and “elevated” cardiovascular disease risk), with a subsequently lower level of cognitive function (mean ACE-III score = 91). Taken together it is likely that the recruitment of a highly active, high-performing sample in the current study contributed to the largely null findings.

Another relevant consideration may be the difference in measures of time use. Fanning et al. (2017) measured waking time-use behaviours (i.e., sedentary behaviour, LPA and MVPA) using accelerometry, but measured sleep duration using self-report (PSQI). This may have resulted in under- or over-estimation of sleep duration. Additionally, Wei et al. (2021) measured time use using the Global Physical Activity Questionnaire, and only included MVPA, walking/bicycling, sedentary behaviour and sleep (duration value obtained during interviews) in their regression models. It is possible both studies did not capture 24-h time use in its entirety.

Finally, two of the previous studies used different measures of cognitive function compared to the current study, namely task-switching and working memory paradigms (Fanning et al., 2017), and tests of memory (CERAD word learning test), language (animal fluency), executive function/processing speed (digit symbol substitution test) and global cognition (composite z-score of all tests) (Wei et al., 2021). It is acknowledged that using scores from single cognitive tests may exhibit a higher level of variability in cognitive function, and conversely, creating composite scores containing multiple tests may mask important differences in individual component scores. Although the composites created for this study were guided by a cognitive domain framework, the number of scores contributing to each composite varied (one for global cognition; one for long-term memory; 4 for short-term memory, 5 for executive function; 4 for processing speed). The true variability of cognitive test scores may have been weakened by creating large composites.

### Sedentary behaviour context, time use and cognitive function

A secondary aim of this study was to investigate whether time spent in sedentary behaviours that require low mental engagement, in this instance TV watching, influenced the associations between time-use composition and cognitive function. We found no significant main effect of TV watching on cognitive function, or interaction effect of TV watching on associations between time-use composition and cognitive function. Overall, our non-significant findings do not align with previous studies that have reported positive associations between mentally stimulating sedentary behaviours (i.e., reading and computer use) and cognitive function, and negative associations between passive sedentary behaviours (e.g., TV watching) and cognitive function (Bakrania et al., 2018; Olanrewaju et al., 2020; Nemoto et al., 2022).

Previous studies measuring sedentary behaviour types and cognitive function in older adult populations have predominantly used self-report measures (Kesse-Guyot et al., 2012; Hamer and Stamatakis, 2014; Falck et al., 2017a). Although self-report measures are able to capture the context of sedentary behaviours, accumulation of activities in older adults is often intermittent or unstructured, which can contribute to over-reporting of sedentary behaviours (Falck et al., 2017a). Conversely, objective measures such as accelerometry do not provide information on which types of sedentary behaviour people are engaging in, which in the context of cognitive function, may be an important consideration. These limitations have led to the recommendation that future studies use a combination of both objective and subjective measures of sedentary behaviour (Falck et al., 2017a). To our knowledge, this is the first study to combine subjective and device-based measures of sedentary behaviour in the same analytical model against cognitive function in older adults. This is also the first study to do so using a CoDA approach, whereby all time-use behaviours are controlled for. It is likely that our methodological approach contributed to the contradictory findings of this study, as most previous studies have not controlled for other time-use behaviours.

### Sleep quality, time use and cognitive function

We did not detect a significant interaction between 24-h time-use composition and sleep quality in association with any of the cognitive measures. Although this interaction has never been directly tested previously, findings from some studies have suggested that perceived sleep quality is important to consider when exploring the relationship between physical activity, sleep duration and cognitive outcomes (Nakakubo et al., 2017; Yuan et al., 2020; Sewell et al., 2023). The lack of evidence supporting this relationship in the current study may be due to several factors. First, the measure used to capture sleep quality in this study relied on a single item measure, chosen to avoid collinearity between total sleep duration measures from the PSQI and accelerometry measures. Second, most participants in this sample rated their sleep quality as “fairly good” (54%) or “very good” (27%), whilst only 17% of participants reported “fairly bad” and 2% reported “very bad” sleep quality. Therefore, over three quarters of participants were classified as having “good” sleep quality in final analyses. This suggests that either, most of the sample had good sleep quality, or that our single-item measure did not capture the true variability in sleep quality. Future studies may benefit from capturing sleep quality or disturbances using objective measures such as accelerometry or polysomnography, to better understand the impact of sleep quality on the associations between time-use composition and cognitive outcomes. Additionally, future studies should consider exploring additional measures such as sleep stage duration, sleep efficiency, and spindle activity, which were identified as predictors of cognitive function in older adults in a recent study (Djonlagic et al., 2021).

### Physical activity context, time use and cognitive function

Although there is some evidence to suggest that recreational physical activity is more beneficial for cognitive function compared to active transport or occupational physical activity (Phansikar et al., 2019), few studies have investigated this in older adults. We found a significant main effect of spending no time in recreational physical activity (relative to >30 min per day) with worse long-term memory performance. However, we did not find an interaction between time-use composition and the amount of recreational physical activity with any cognitive outcomes. This may be due in part to the low proportion of participants who reported engaging in recreational physical activity: over half (54%) of participants reported engaging in no recreational physical activity, and only one quarter (27%) engaged in more than 30 min per day. Given that the average time per day spent in MVPA and LPA (as measured by accelerometry) was ∼1.5 and ∼3 h respectively, this may suggest that our participants accumulated their physical activity *via* other modalities, including active transport (walking, cycling), occupational, or household (i.e., gardening, sweeping). This aligns with evidence that older adults >65 years of age generally prefer walking as their mode of physical activity, whilst older adults aged >74 years more often prefer exercising in social contexts, such as recreational group fitness classes (Amireault et al., 2019). Thus, future studies in this age group should consider physical activity contexts beyond recreational PA.

### Strengths and limitations

To our knowledge, this study was the first to investigate the associations between 24-h time use and cognitive function in older adults using device-based measures (accelerometry) to capture time use, and to additionally consider the role of the context and quality of time-use behaviours in these relationships. A wide range of reliable cognitive tests were used to examine whether 24-h time use is associated with cognitive function across a range of domains. We took a conservative approach to interpreting the findings by adjusting for false discovery rate which is not commonly done in exploratory studies (Jafari and Ansari-Pour, 2019). However, there are several limitations to consider. First, despite efforts to recruit participants who represented a variety of activity patterns and dietary patterns (for the ACTIVate study), the final sample were highly active, highly educated, and subsequently achieved high scores on the cognitive assessments (i.e., average ACE-III score = 95). Second, the cross-sectional nature of this study does not allow causal inferences to be made about the relationships between variables. Third, the CANTAB tests took approximately 40 min in total and were conducted in the same order for all participants, so it may be possible that those conducted toward the end of the battery were impacted by fatigue. However, given the consistency in order of test completion, these effects would be systematic across the cohort. Finally, due to study constraints there were inconsistencies in the days being recalled during the MARCA, which resulted in some participants including a weekend day in their 2-day recall, and others only recalling weekdays. This may have contributed to variability in recall of recreational physical activity and TV watching.

### Conclusions and future directions

Our study identified independent correlations between time use variables and cognitive outcomes, however, linear regression models found no significant associations between 24-h time-use composition and cognitive function outcomes, and no significant interactions between TV watching time, recreational physical activity engagement or sleep quality and time-use composition for any cognitive outcomes. Together these findings highlight the importance of considering all activities across the 24-h day against cognitive function in older adults. Future studies should consider investigating these relationships longitudinally to uncover temporal effects.

## Data availability statement

The raw data supporting the conclusions of this article will be made available by the authors, without undue reservation.

## Ethics statement

The studies involving human participants were reviewed and approved by University of South Australia and University of Newcastle Human Research Ethics Committees. The patients/participants provided their written informed consent to participate in this study.

## Author contributions

MM contributed to the study design, data analysis, interpretation of results, writing, and drafting of the manuscript. DD and TS contributed to the data analysis, interpretation of results, and drafting of the manuscript. AW contributed to the study design, data collection, interpretation of results, and drafting of the manuscript. TO, FK, and HK contributed to the study design, interpretation of results, and drafting of the manuscript. MH contributed to the study design, data collection, and drafting of the manuscript. JD and MG contributed to the interpretation of results and drafting of the manuscript. AS contributed to study design, data collection, interpretation of results, and drafting of the manuscript. All authors contributed to the article and approved the submitted version.
