# Corrigendum: Twenty-four-hour time-use composition and cognitive function in older adults: cross-sectional findings of the ACTIVate study

**DOI:** 10.3389/fnhum.2023.1221303

**Published:** 2023-06-09

**Authors:** Maddison L. Mellow, Dorothea Dumuid, Alexandra T. Wade, Ty Stanford, Timothy S. Olds, Frini Karayanidis, Montana Hunter, Hannah A. D. Keage, Jillian Dorrian, Mitchell R. Goldsworthy, Ashleigh E. Smith

**Affiliations:** ^1^Alliance for Research in Exercise, Nutrition and Activity, Allied Health and Human Performance, University of South Australia, Adelaide, SA, Australia; ^2^Functional Neuroimaging Laboratory, School of Psychological Sciences, The University of Newcastle, Newcastle, NSW, Australia; ^3^Healthy Minds Research Program, Hunter Medical Research Institute (HMRI), Newcastle, NSW, Australia; ^4^Behaviour-Brain-Body Research Centre, Justice and Society, University of South Australia, Adelaide, SA, Australia; ^5^Lifespan Human Neurophysiology Group, School of Biomedicine, University of Adelaide, Adelaide, SA, Australia; ^6^Hopwood Centre for Neurobiology, Lifelong Health Theme, South Australian Health and Medical Research Institute (SAHMRI), Adelaide, SA, Australia

**Keywords:** time use, cognitive function, ageing, sleep, sedentary behaviour, physical activity

In the published article, there were several errors in Table 2 as published. The mean ACE-III scores presented for Adelaide participants, Newcastle participants and the total sample were presented incorrectly due to errors in the calculation of ACE-III scores. The corrected Table 2 and its caption appear below.

**Table 2:** Participant demographics.

**Variable**	**Level**	**Adelaide (*n* = 207)**	**Newcastle (*n* = 177)**	**Total (*n* = 384)**
Age		65.6 ± 2.8	65.4 ± 3.2	65.5 ± 3.0
Sex	Female	165	98	263
	Male	42	79	121
Education (years)		16.3 ± 3.3	16.7 ± 3.2	16.5 ± 3.2
Smoking status	Current smoker	84 (41%)	59 (33%)	143 (37%)
	Previous smoker	7 (3%)	0 (0%)	7 (2%)
	Never smoked	116 (56%)	118 (67%)	234 (61%)
Device-measured PA levels (min/day)	MVPA	91 ± 46	86 ± 47	89 ± 47
	LPA	178 ± 48	178 ± 52	178 ± 50
	SB	657 ± 90	682 ± 87	668 ± 90
	Sleep	513 ± 59	492 ± 53	503 ± 57
Sleep quality rating	Good	165 (79.7%)	149 (84.2%)	314 (81.6%)
Bad	42 (20.3%)	28 (15.8%)	70 (18.8%)
Recreational PA (min/day)	“None”	0	0	0
	“ < 30”	21 ± 8	17 ± 8	19 ± 8
	“>30”	80 ± 49	81 ± 59	80 ± 53
TV watching (min/day)	High	223 ± 48	225 ± 67	224 ± 59
	Medium	128 ± 21	123 ± 17	126 ± 20
	Low	43 ± 28	47 ± 33	44 ± 30
ACE-III score		95.8 ± 3.1	94.2 ± 3.9	95.1 ± 3.6

Values are presented as either mean ± SD for numeric variables or count (percentage) for categorical variables. Recreational physical activity (PA) and TV watching data are presented as the mean ± SD minutes per day spent in respective activities.

MVPA, moderate-vigorous physical activity; LPA, light physical activity; SB, sedentary behaviour; PME, perceived mental effort; ACE-III, Addenbrooke's Cognitive Examination III.

In the published article, there were several errors in Table 3 as published. Correlations between executive function and other outcomes were incorrect due to an error in the calculation of the executive function composite score, and correlations between ACE-III score and other outcomes were incorrect due to minor errors in the calculation of the ACE-III scores. The corrected Table 3 and its caption appear below.

**Table 3:** Pairwise correlations between time-use variables and cognitive outcomes.

	**ACE-III**	**Long-term memory**	**Short-term memory**	**Executive function**	**Processing speed**	**Sleep (min)**	**SB (min)**	**LPA (min)**
Long-term memory	0.22^**^							
Short-term memory	0.31^**^	0.57^**^						
Executive function	0.16^**^	0.22^**^	0.21^**^					
Processing speed	0.09	0.06	0.03	0.03				
Sleep (min)	0.00	−0.11^*^	−0.06	0.02	0.00			
SB (min)	0.00	0.05	0.05	0.01	−0.13^*^	−0.45^**^		
LPA (min)	−0.04	0.02	−0.01	−0.05	0.06	−0.16^**^	−0.68^**^	
MVPA (min)	0.03	0.04	0.01	0.01	0.17^**^	−0.14^**^	−0.65^**^	0.43^**^

Data are presented as Pearson correlation coefficients (r). Bold denotes that the p-value is statistically significant.

* Denotes p-values ≤ 0.05.

** Denotes p-values ≤ 0.01.

ACE-III, Addenbrooke's Cognitive Examination III; SB, sedentary behaviour; LPA, light physical activity; MVPA, moderate to vigorous physical activity.

In the published article, there were several errors in Table 4 as published. ANOVA type II *F*-test outcomes for executive function were incorrect due to an error in the calculation of the executive function composite score, and ANOVA type II *F*-test outcomes for ACE-III score were incorrect due to errors in the calculation of ACE-III scores. The corrected Table 4 and its caption appear below.

**Table 4:** Statistical output of ANOVA type II *F*-tests for cognitive outcomes.

	**Global cognition**			**Long-term memory**			**Short-term memory**			**Executive function**			**Processing speed**		
	*F*_(n, d)_	***p*** **-value**	***adj.p***	*F*_(n, d)_	***p*** **-value**	***adj.p***	*F*_(n, d)_	***p*** **-value**	***adj.p***	*F*_(n, d)_	***p*** **-value**	***adj.p***	*F*_(n, d)_	***p*** **-value**	***adj.p***
Age	0.68_(1, 372)_	0.41	0.54	1.18_(1, 354)_	0.28	0.32	2.52_(1, 293)_	0.11	0.31	29.16_(1, 357)_	**< 0.01**	< 0.01^*^	7.74_(1, 359)_	**≤0.01**	0.05
Sex	1.20_(1, 372)_	0.27	0.44	3.98_(1, 354)_	0.05	0.13	0.05_(1, 293)_	0.81	0.81	15.73_(1, 357)_	**< 0.01**	< 0.01^*^	0.17_(1, 359)_	0.68	0.96
Site	15.20_(1, 372)_	**≤0.01**	≤0.01^*^	5.19_(1, 354)_	**0.02**	0.09	6.14_(1, 293)_	**0.01**	0.11	0.70_(1, 357)_	0.40	0.49	3.05_(1, 359)_	0.08	0.24
Smoking status	0.75_(2, 372)_	0.47	0.54	2.28_(2, 354)_	0.10	0.17	2.08_(2, 293)_	0.13	0.31	0.68_(2, 357)_	0.51	0.51	0.08_(2, 359)_	0.92	0.96
Education (years)	16.44_(1, 372)_	**≤0.01**	≤0.01^*^	2.13_(1, 354)_	0.15	0.19	1.13_(1, 293)_	0.29	0.45	3.81_(1, 357)_	0.05	0.10	0.10_(1, 359)_	0.75	0.96
Sleep quality	3.43_(3, 372)_	0.06	0.13	0.04_(3, 354)_	0.85	0.85	0.62_(3, 293)_	0.43	0.49	0.64_(3, 357)_	0.43	0.49	0.30_(3, 359)_	0.58	0.96
TV time (min/day)	2.78_(2, 372)_	0.06	0.13	2.29_(2, 354)_	0.10	0.16	1.88_(2, 293)_	0.15	0.31	0.97_(2, 357)_	0.38	0.49	0.03_(2, 359)_	0.97	0.97
Recreational PA (min/day)	0.36_(2, 372)_	0.70	0.70	5.15_(2, 354)_	**≤0.01**	0.05^*^	1.09_(2, 293)_	0.34	0.45	3.09_(2, 357)_	**0.05**	0.10	1.33_(2, 359)_	0.27	0.60
Time-use composition	–	–	–	–	–	–	–	–	–	–	–	–	2.87_(3, 359)_	**0.04**	0.16

F_(*n, d*)_ = F statistic, and numerator and denominator degrees of freedom; adj.p = p-value adjusted for false discovery rate. Bold denotes statistical significance (p ≤ 0.05).

* Denotes p-values that remained significant after false discovery rate adjustment.

“–” Denotes variables that were not included in final models for respective cognitive outcomes. Interaction terms (for sleep quality, recreational PA or TV watching) were not included in final models for any cognitive outcomes and therefore are not listed in this table.

In the published article, there was an error in Supplementary Material 3 (Table 1). The linear regression output for the global cognition outcome was based on incorrect data due to error in calculation of ACE-III total scores. The correct material appears below.

***Global cognition***

Final model: global cognition ~ age + sex + site + smoking status + education + sleep quality + TV watching time + recreational physical activity

**Table:** 

**Variable**	**Level**	**Estimate**	**Std. Error**	**t value**	**p-value**
[intercept]		93.94	4.08	23.03	< 0.01
Age		−0.05	0.06	−0.83	0.41
Sex	Female	0.43	0.39	1.10	0.27
Site	Newcastle	−1.43	0.37	−3.90	< 0.01
Smoking status	Never smoked	−0.05	0.36	−0.14	0.88
	Previous smoker	1.57	1.33	1.18	0.24
Education (years)		0.22	0.05	4.06	< 0.01
Sleep quality	“Good”	0.85	0.46	1.85	0.06
TV watching time	Low	1.01	0.43	2.34	0.02
	Medium	0.40	0.43	0.93	0.35
Recreational physical activity	Under 30 minutes	0.09	0.52	0.18	0.86
	Zero	−0.25	0.41	−0.60	0.55

In the published article, there was an error in Supplementary Material 3 (Table 4). The linear regression output for the executive function outcome was based on incorrect data (due to error in calculation of executive function composite score). The correct material appears below.

***Executive function***

Final model: executive function ~ age + sex + site + smoking status + education + sleep quality + TV watching time + recreational physical activity

**Table:** 

**Variable**	**Level**	**Estimate**	**Std. Error**	**t value**	**p-value**
[intercept]		2.98	0.59	5.08	< 0.01
Age		−0.05	0.01	−5.40	< 0.01
Sex	Female	−0.23	0.06	−3.97	< 0.01
Site	Newcastle	0.04	0.05	0.84	0.40
Smoking status	Never smoked	−0.02	0.05	−0.37	0.71
	Previous smoker	−0.22	0.19	−1.15	0.25
Education (years)		0.16	0.01	1.95	0.05
Sleep quality	“Good”	0.05	0.07	0.80	0.43
TV watching time	Low	−0.06	0.06	−0.91	0.37
	Medium	−0.09	0.06	−1.38	0.17
Recreational physical activity	Under 30 minutes	−0.03	0.08	−0.44	0.66
	Zero	−0.14	0.06	−2.31	0.02

In the published article, a number of statements were made about global cognition and executive functions outcomes which were based on incorrect data. We have now corrected the data and have subsequently altered a number of text sections to reflect the correct findings for global cognition and executive functions outcomes.

A correction has been made to **Section 3.1**, **Participant demographics**, paragraph 1. This sentence previously stated:

“A number of participants were removed from each analysis due to missing cognitive data: total samples for each cognitive outcome included: *n* = 372 for global cognition; *n* = 292 for short-term memory; *n* = 353 for long-term memory; *n* = 356 for executive functions; *n* = 358 for processing speed.”

The corrected sentence appears below:

“A number of participants were removed from each analysis due to missing cognitive data: total samples for each cognitive outcome included: *n* = 384 for global cognition; *n* = 292 for short-term memory; *n* = 353 for long-term memory; *n* = 369 for executive functions; *n* = 358 for processing speed”.

A correction has been made to **Section 3.2.1**, **Pairwise correlations**, paragraph 1. This sentence previously stated:

“Pearson correlation coefficients revealed that time spent in sleep was negatively correlated with long term memory (*r* = −0.11, *p* = 0.03) and executive function (*r* = −0.12, *p* = 0.02), time spent in sedentary behaviour was negatively correlated with processing speed (*r* = −0.13, *p* = 0.01), and time spent in MVPA was positively correlated with processing speed (*r* = 0.17, *p* < 0.01).”

The corrected sentence appears below:

“Pearson correlation coefficients revealed that time spent in sleep was negatively correlated with long term memory (*r* = −0.11, *p* = 0.03), time spent in sedentary behaviour was negatively correlated with processing speed (*r* = −0.13, *p* = 0.01), and time spent in MVPA was positively correlated with processing speed (*r* = 0.17, *p* < 0.01).”

A correction has been made to **Section 3.2.2**, **Linear regression models**, paragraph 1. This sentence previously stated:

“Additionally, several covariates were significantly associated with cognitive outcomes: older age was associated with better executive function (β = 0.02) and slower processing speed (β = −0.38); site was negatively associated with global cognition (β = −1.01) and positively associated with long-term memory (β = 0.25) and short-term memory (β = 0.17) (i.e., participants from Newcastle had lower global cognition scores and higher long-term and short-term memory scores than Adelaide); higher education (years) was associated with better global cognition (β = 0.18); and engaging in no recreational physical activity (relative to >30 min) was associated with poorer long-term memory (β = −0.38).”

The corrected sentence appears below:

“Additionally, several covariates were significantly associated with cognitive outcomes: older age was associated with poorer executive function (β = −0.05) and slower processing speed (β = −0.04); site was negatively associated with global cognition (β = −1.43) and positively associated with long-term memory (β = 0.25) and short-term memory (β = 0.17) (i.e., participants from Newcastle had lower global cognition scores and higher long-term and short-term memory scores than Adelaide); higher education (years) was associated with better global cognition (β = 0.22); sex (being female) was negatively associated with executive function (β = −0.23) and engaging in no recreational physical activity (relative to >30 min) was associated with poorer long-term memory (β = −0.38).”

A correction has been made to **Section 3.2.2**, **Linear regression models**, paragraph 2. This sentence previously stated:

“After false discovery rate adjustment, none of the associations between 24-h time-use composition and cognitive function outcomes were statistically significant. Associations between age and processing speed, recreational physical activity and long-term memory, as well as education, site and global cognition, remained significant. Unadjusted and adjusted *p*-values for all variables across each cognitive outcome are displayed in Table 4. Linear regression outputs for each cognitive outcome can be found in Supplementary material 3”.

The corrected sentence appears below:

“After false discovery rate adjustment, none of the associations between 24-h time-use composition and cognitive function outcomes were statistically significant. Associations between age and executive functions, recreational physical activity and long-term memory, sex and executive functions, as well as education, site and global cognition, remained significant. Unadjusted and adjusted *p*-values for all variables across each cognitive outcome are displayed in Table 4. Linear regression outputs for each cognitive outcome can be found in **Supplementary material 3**”.

A correction has been made to **Section 4.1**, **24-hour time use composition and cognitive function in older adults**, paragraph 1. This sentence previously stated:

“We initially explored pairwise correlations to understand the independent and unadjusted associations between time use variables and cognitive outcomes, in which we found that sleep was negatively correlated with long-term memory and executive function, sedentary behaviour was negatively correlated with processing speed, and MVPA was positively correlated with processing speed. Subsequently, after adjusting for demographic and health factors that are associated with risk of dementia (age, sex, education, smoking status), linear regression models demonstrated 24-h time-use composition was significantly associated with processing speed, but there were no associations with global cognition, long-term memory, short-term memory or executive function.”

The corrected sentence appears below:

“We initially explored pairwise correlations to understand the independent and unadjusted associations between time use variables and cognitive outcomes, in which we found that sleep was negatively correlated with long-term memory, sedentary behaviour was negatively correlated with processing speed, and MVPA was positively correlated with processing speed. Subsequently, after adjusting for demographic and health factors that are associated with risk of dementia (age, sex, education, smoking status), linear regression models demonstrated 24-h time-use composition was significantly associated with processing speed, but there were no associations with global cognition, long-term memory, short-term memory or executive function.”

The authors apologize for these errors and state that these do not change the scientific conclusions of the article in any way. The original article has been updated.

