# Supplementary material for: Twenty-four-hour time-use composition and cognitive function in older adults: cross-sectional findings of the ACTIVate study
Source: Front Hum Neurosci. 2022 Nov 24;16:1051793. doi: 10.3389/fnhum.2022.1051793 (PMC9729737; doi:10.3389/fnhum.2022.1051793)
Supplement: Supplementary file 1 [file Data_Sheet_1.docx]

Supplementary Material

Supplementary Material 1 . Recreational physical activity categorization

The following list highlights the types of activities that were categorized as recreational physical activity (superdomain = ‘Physical Activity’)within the Multimedia Activity Recall for Children and Adults (MARCA) assessment.

| **Macrodomain** | **Microdomain** | **Activity** |
| --- | --- | --- |
| play | animals | playing with animals - sitting |
| play | animals | playing with animals - walk/run - light |
| play | animals | playing with animals - walk/run - medium |
| play | animals | playing with animals - walk/run - hard |
| play | games | quoits |
| play | games | totem tennis - light |
| play | games | totem tennis - medium |
| play | games | totem tennis - hard |
| play | games | hacky sack - light |
| play | games | hacky sack - medium |
| play | games | hacky sack - hard |
| play | games | playing catch - light |
| play | games | playing catch - medium |
| play | games | playing catch - hard |
| play | games | mini golf or putt putt |
| play | games | paddleball - light |
| play | games | paddleball - medium |
| play | games | paddleball - hard |
| play | games | frisbee (general) - light |
| play | games | frisbee (general) - medium |
| play | games | frisbee (general) - hard |
| play | games | frisbee (ultimate) - light |
| play | games | frisbee (ultimate) - medium |
| play | games | frisbee (ultimate) - hard |
| play | games | pool/billiards/snooker |
| play | games | table tennis - light |
| play | games | table tennis - medium |
| play | games | table tennis - hard |
| play | games | tenpin bowling |
| play | games | juggling |
| play | games | darts |
| sport | dance | dancing (general) - light |
| sport | dance | dancing (general) - medium |
| sport | dance | dancing (general) - hard |
| sport | dance | ballet - light |
| sport | dance | ballet - medium |
| sport | dance | ballet - hard |
| sport | dance | tap/jazz dancing - light |
| sport | dance | tap/jazz dancing - medium |
| sport | dance | tap/jazz dancing - hard |
| sport | gym | lifting weights - light |
| sport | gym | lifting weights - medium |
| sport | gym | lifting weights - hard |
| sport | gym | gym - stationary rowing - light |
| sport | gym | gym - stationary rowing - medium |
| sport | gym | gym - stationary rowing - hard |
| sport | gym | gym - elliptical trainer - light |
| sport | gym | gym - elliptical trainer - medium |
| sport | gym | gym - elliptical trainer - hard |
| sport | gym | fitball exercises - light |
| sport | gym | fitball exercises - medium |
| sport | gym | fitball exercises - hard |
| sport | gym | gym - stationary cycling/bike - light |
| sport | gym | gym - stationary cycling/bike - medium |
| sport | gym | gym - stationary cycling/bike - hard |
| sport | gym | gym - push ups/sit ups/pull ups etc - light |
| sport | gym | gym - push ups/sit ups/pull ups etc - medium |
| sport | gym | gym - push ups/sit ups/pull ups etc - hard |
| sport | gym | gym - circuit training with minimal rest |
| sport | gym | gym - ski machine - light |
| sport | gym | gym - ski machine - medium |
| sport | gym | gym - ski machine - hard |
| sport | gym | gym - stair machine - light |
| sport | gym | gym - stair machine - medium |
| sport | gym | gym - stair machine - hard |
| sport | gym | skipping/jump rope - light |
| sport | gym | skipping/jump rope - medium |
| sport | gym | skipping/jump rope - hard |
| sport | gym | aerobics/health hustle - light |
| sport | gym | aerobics/health hustle - medium |
| sport | gym | aerobics/health hustle - hard |
| sport | gym | light home exercises (eg back exercises) |
| sport | gym | stretching exercises |
| sport | gym | tai chi/yoga |
| sport | gym | gymnastics - light |
| sport | gym | gymnastics - medium |
| sport | gym | gymnastics - hard |
| sport | gym | karate/martial arts/judo/kick boxing - light |
| sport | gym | karate/martial arts/judo/kick boxing - medium |
| sport | gym | karate/martial arts/judo/kick boxing - hard |
| sport | gym | using a punching bag - light |
| sport | gym | using a punching bag - medium |
| sport | gym | using a punching bag - hard |
| sport | miscellaneous sports | rockclimbing - light |
| sport | miscellaneous sports | rockclimbing - medium |
| sport | miscellaneous sports | rockclimbing - hard |
| sport | miscellaneous sports | archery |
| sport | miscellaneous sports | horseback riding - light |
| sport | miscellaneous sports | horseback riding - medium |
| sport | miscellaneous sports | horseback riding - hard |
| sport | miscellaneous sports | horse racing - galloping |
| sport | miscellaneous sports | horse racing - trotting |
| sport | miscellaneous sports | horse racing - walking |
| sport | miscellaneous sports | golf - light |
| sport | miscellaneous sports | golf - medium |
| sport | miscellaneous sports | golf - hard |
| sport | partner sports | racquetball - light |
| sport | partner sports | racquetball - medium |
| sport | partner sports | racquetball - hard |
| sport | partner sports | squash - light |
| sport | partner sports | squash - medium |
| sport | partner sports | squash - hard |
| sport | partner sports | tennis (court) - light |
| sport | partner sports | tennis (court) - medium |
| sport | partner sports | tennis (court) - hard |
| sport | partner sports | badminton - light |
| sport | partner sports | badminton - medium |
| sport | partner sports | badminton - hard |
| sport | partner sports | croquet |
| sport | snow sports | snowboarding - light |
| sport | snow sports | snowboarding - medium |
| sport | snow sports | snowboarding - hard |
| sport | snow sports | snowshoeing - light |
| sport | snow sports | snowshoeing - medium |
| sport | snow sports | snowshoeing - hard |
| sport | snow sports | ice skating - light |
| sport | snow sports | ice skating - medium |
| sport | snow sports | ice skating - hard |
| sport | snow sports | skiing (cross-country) - light |
| sport | snow sports | skiing (cross-country) - medium |
| sport | snow sports | skiing (cross-country) - hard |
| sport | snow sports | skiing (downhill) - light |
| sport | snow sports | skiing (downhill) - medium |
| sport | snow sports | skiing (downhill) - hard |
| sport | snow sports | speed skating (competitive) - light |
| sport | snow sports | speed skating (competitive) - medium |
| sport | snow sports | speed skating (competitive) - hard |
| sport | snow sports | bobsled toboggan luge - light |
| sport | snow sports | bobsled toboggan luge - medium |
| sport | snow sports | bobsled toboggan luge - hard |
| sport | snow sports | curling - light |
| sport | snow sports | curling - medium |
| sport | snow sports | curling - hard |
| sport | snow sports | hockey (ice) - light |
| sport | snow sports | hockey (ice) - medium |
| sport | snow sports | hockey (ice) - hard |
| sport | team sports | baseball - light |
| sport | team sports | baseball - medium |
| sport | team sports | baseball - hard |
| sport | team sports | basketball - light |
| sport | team sports | basketball - medium |
| sport | team sports | basketball - hard |
| sport | team sports | cricket - light |
| sport | team sports | cricket - medium |
| sport | team sports | cricket - hard |
| sport | team sports | football (Australian/Gaelic/American) - light |
| sport | team sports | football (Australian/Gaelic/American) - medium |
| sport | team sports | football (Australian/Gaelic/American) - hard |
| sport | team sports | rugby league - light |
| sport | team sports | rugby league - medium |
| sport | team sports | rugby league - hard |
| sport | team sports | rugby union - light |
| sport | team sports | rugby union - medium |
| sport | team sports | rugby union - hard |
| sport | team sports | soccer (field/indoor) - light |
| sport | team sports | soccer (field/indoor) - medium |
| sport | team sports | soccer (field/indoor) - hard |
| sport | team sports | European handball (team) - light |
| sport | team sports | European handball (team) - medium |
| sport | team sports | European handball (team) - hard |
| sport | team sports | hockey (field) - light |
| sport | team sports | hockey (field) - medium |
| sport | team sports | hockey (field) - hard |
| sport | team sports | lacrosse - light |
| sport | team sports | lacrosse - medium |
| sport | team sports | lacrosse - hard |
| sport | team sports | lawn bowls |
| sport | team sports | netball - light |
| sport | team sports | netball - medium |
| sport | team sports | netball - hard |
| sport | team sports | coaching (eg football/soccer/basketball) |
| sport | team sports | softball or t-ball - light |
| sport | team sports | softball or t-ball - medium |
| sport | team sports | softball or t-ball - hard |
| sport | team sports | volleyball (court) - light |
| sport | team sports | volleyball (court) - medium |
| sport | team sports | volleyball (court) - hard |
| sport | team sports | volleyball (beach) - light |
| sport | team sports | volleyball (beach) - medium |
| sport | team sports | volleyball (beach) - hard |
| sport | team sports | touch football - light |
| sport | team sports | touch football - medium |
| sport | team sports | touch football - hard |
| sport | track and field | athletics (track and field): jumping - light |
| sport | track and field | athletics (track and field): jumping - medium |
| sport | track and field | athletics (track and field): jumping - hard |
| sport | track and field | athletics (track and field): hurdles steeplechase - light |
| sport | track and field | athletics (track and field): hurdles steeplechase - medium |
| sport | track and field | athletics (track and field): hurdles steeplechase - hard |
| sport | track and field | athletics (track and field): throwing - light |
| sport | track and field | athletics (track and field): throwing - medium |
| sport | track and field | athletics (track and field): throwing - hard |
| sport | track and field | orienteering - light |
| sport | track and field | orienteering - medium |
| sport | track and field | orienteering - hard |
| sport | track and field | running/jogging - light |
| sport | track and field | running/jogging - medium |
| sport | track and field | running/jogging - hard |
| sport | track and field | race walking - light |
| sport | track and field | race walking - medium |
| sport | track and field | race walking - hard |
| sport | water sports | sailboard/windsurfing - light |
| sport | water sports | sailboard/windsurfing - medium |
| sport | water sports | sailboard/windsurfing - hard |
| sport | water sports | water skiing - light |
| sport | water sports | water skiing - medium |
| sport | water sports | water skiing - hard |
| sport | water sports | jetskiing |
| sport | water sports | sailing/boating - light |
| sport | water sports | sailing/boating - medium |
| sport | water sports | sailing/boating - hard |
| sport | water sports | rowing/canoeing - light |
| sport | water sports | rowing/canoeing - medium |
| sport | water sports | rowing/canoeing - hard |
| sport | water sports | kayaking - light |
| sport | water sports | kayaking - medium |
| sport | water sports | kayaking - hard |
| sport | water sports | skindiving (SCUBA) - light |
| sport | water sports | skindiving (SCUBA) - medium |
| sport | water sports | skindiving (SCUBA) - hard |
| sport | water sports | snorkeling - light |
| sport | water sports | snorkeling - medium |
| sport | water sports | snorkeling - hard |
| sport | water sports | surfing (body or board) - light |
| sport | water sports | surfing (body or board) - medium |
| sport | water sports | surfing (body or board) - hard |
| sport | water sports | swimming laps - light |
| sport | water sports | swimming laps - medium |
| sport | water sports | swimming laps - hard |
| sport | water sports | whitewater rafting - light |
| sport | water sports | whitewater rafting - medium |
| sport | water sports | whitewater rafting - hard |
| sport | water sports | swimming (playing in pool) - light |
| sport | water sports | swimming (playing in pool) - medium |
| sport | water sports | swimming (playing in pool) - hard |
| sport | water sports | water aerobics - light |
| sport | water sports | water aerobics - medium |
| sport | water sports | water aerobics - hard |
| sport | water sports | water polo - light |
| sport | water sports | water polo - medium |
| sport | water sports | water polo - hard |
| sport | water sports | fishing - sitting |
| sport | water sports | fishing - standing |
| sport | water sports | fishing - in the water with waders |

Supplementary Material 2. Pre-processing protocol for cognitive measures.

The following text outlines the steps taken to pre-process cognitive data (from raw data to cognitive composites).

**1.1 Pre-processing of cognitive measures:**

1) Exclude participants who were ≥50% inaccurate on CANTAB tests based on following criteria:

**Verbal recognition memory**: <18/36 on delayed recognition total correct (VRMDRTC); <18/36 on immediate recognition total correct (VRMIRTC)

**Paired Associates Learning:**>35/70 on total errors adjusted (PALTEA)

**Multitasking Test**: >80/160 on total incorrect responses (MTTTIC)

**Reaction Time**: >15/30 on simple total error score (RTISES); >15/30 on 5-choice total error score (RTIFES)

**One Touch Stockings of Cambridge**: >4/7 on mean choices to correct (OTSMCC)

2) Reverse-score outcome measures so that higher scores = better performance for all measures. The following outcome measures need to be reverse-scored:

- Paired Associates Learning total errors (adjusted) (PALTEA)
- Multitasking Test total incorrect responses (MTTTIC)
- Multitasking Test median response latency multitasking cost (MTTMTCMD)
- Multitasking Test median response latency incongruency cost (MTTICMD)
- One Touch Stockings of Cambridge median latency to first choice (OTSMDLFC)
- Reaction Time simple median reaction time (RTISMDRT)
- Reaction Time 5-choice median reaction time (RTIFMDRT)
- Reaction Time simple median movement time (RTISMDMT)
- Reaction Time 5-choice median movement time (RTIFMDMT)

3) Convert raw scores to z-scores for each outcome measure

4) Combine z-scores into cognitive composites:

Long Term Memory = VRMDRTCz

Short Term Memory = (PALTEAz + PALFAMSz + VRMIRTCz + VRMFRDSz) / 4

Executive Functions = (MTTICMDz + MTTMTCMDz + MTTTICz + OTSPSFCz + OTSMDLFCz) / 5

Processing Speed = (RTISMDRTz + RTISMDMTz + RTIFMDRTz + RTIFMDMTz) / 4

***note: if participants are missing any data within the composite (i.e., missing MTTICMD data for executive function), the composite is incomplete and won’t be calculated.

Supplementary Material 3. Linear regression model outputs

The following tables contain regression outputs from final models for each cognitive outcome. Presented p-values are unadjusted (as only p-values from ANOVA outputs were adjusted for false discovery rate).

**Global cognition**

Final model: global cognition ~ age + sex + site + smoking status + education + sleep quality + TV watching time + recreational physical activity

| **Variable** | **Level** | **Estimate** | **Std. Error** | **t value** | **p-value** |
| --- | --- | --- | --- | --- | --- |
| [intercept] |  | 93.94 | 4.08 | 23.03 | <0.01 |
| Age |  | -0.05 | 0.06 | -0.83 | 0.41 |
| Sex | Female | 0.43 | 0.39 | 1.10 | 0.27 |
| Site | Newcastle | -1.43 | 0.37 | -3.90 | <0.01 |
| Smoking status | Never smoked | -0.05 | 0.36 | -0.14 | 0.88 |
|  | Previous smoker | 1.57 | 1.33 | 1.18 | 0.24 |
| Education (years) |  | 0.22 | 0.05 | 4.06 | <0.01 |
| Sleep quality | “Good” | 0.85 | 0.46 | 1.85 | 0.06 |
| TV watching time | Low | 1.01 | 0.43 | 2.34 | 0.02 |
|  | Medium | 0.40 | 0.43 | 0.93 | 0.35 |
| Recreational physical activity | Under 30 minutes | 0.09 | 0.52 | 0.18 | 0.86 |
|  | Zero | -0.25 | 0.41 | -0.60 | 0.55 |

**Long-term memory**

Final model: long-term memory ~ age + sex + site + smoking status + education + sleep quality + TV watching time + recreational physical activity

| **Variable** | **Level** | **Estimate** | **Std. Error** | **t value** | **p-value** |
| --- | --- | --- | --- | --- | --- |
| [intercept] |  | 0.85 | 1.19 | 0.72 | 0.47 |
| Age |  | -0.02 | 0.02 | -1.09 | 0.28 |
| Sex | Female | 0.23 | 0.11 | 1.99 | 0.05 |
| Site | Newcastle | 0.25 | 0.11 | 2.28 | 0.02 |
| Smoking status | Never smoked | 0.14 | 0.11 | 1.28 | 0.20 |
|  | Previous smoker | -0.61 | 0.41 | -1.50 | 0.14 |
| Education (years) |  | 0.02 | 0.02 | 1.46 | 0.15 |
| Sleep quality | “Good” | 0.03 | 0.13 | 0.19 | 0.85 |
| TV watching time | Low | -0.09 | 0.13 | -0.75 | 0.45 |
|  | Medium | -0.27 | 0.13 | -2.10 | 0.04 |
| Recreational physical activity | Under 30 minutes | -0.18 | 0.15 | -1.16 | 0.25 |
|  | Zero | -0.38 | 0.12 | -3.15 | <0.01 |

**Short-term memory**

Final model: short-term memory ~ age + sex + site + smoking status + education + sleep quality + TV watching time + recreational physical activity

| **Variable** | **Level** | **Estimate** | **Std. Error** | **t value** | **p-value** |
| --- | --- | --- | --- | --- | --- |
| [intercept] |  | 1.16 | 0.78 | 1.47 | 0.14 |
| Age |  | -0.02 | 0.01 | -1.59 | 0.11 |
| Sex | Female | 0.02 | 0.08 | 0.24 | 0.81 |
| Site | Newcastle | 0.17 | 0.07 | 2.48 | 0.01 |
| Smoking status | Never smoked | -0.07 | 0.07 | -0.99 | 0.33 |
|  | Previous smoker | -0.48 | 0.25 | -1.95 | 0.05 |
| Education (years) |  | 0.01 | 0.01 | 1.07 | 0.29 |
| Sleep quality | “Good” | 0.07 | 0.09 | 0.77 | 0.43 |
| TV watching time | Low | 0.15 | 0.08 | 1.80 | 0.07 |
|  | Medium | 0.02 | 0.08 | 0.24 | 0.81 |
| Recreational physical activity | Under 30 minutes | -0.11 | 0.10 | -1.09 | 0.28 |
|  | Zero | -0.11 | 0.08 | -1.43 | 0.16 |

**Executive function**

Final model: executive function ~ age + sex + site + smoking status + education + sleep quality + TV watching time + recreational physical activity

| **Variable** | **Level** | **Estimate** | **Std. Error** | **t value** | **p-value** |
| --- | --- | --- | --- | --- | --- |
| [intercept] |  | 2.98 | 0.59 | 5.08 | <0.01 |
| Age |  | -0.05 | 0.01 | -5.40 | <0.01 |
| Sex | Female | -0.23 | 0.06 | -3.97 | <0.01 |
| Site | Newcastle | 0.04 | 0.05 | 0.84 | 0.40 |
| Smoking status | Never smoked | -0.02 | 0.05 | -0.37 | 0.71 |
|  | Previous smoker | -0.22 | 0.19 | -1.15 | 0.25 |
| Education (years) |  | 0.16 | 0.01 | 1.95 | 0.05 |
| Sleep quality | “Good” | 0.05 | 0.07 | 0.80 | 0.43 |
| TV watching time | Low | -0.06 | 0.06 | -0.91 | 0.37 |
|  | Medium | -0.09 | 0.06 | -1.38 | 0.17 |
| Recreational physical activity | Under 30 minutes | -0.03 | 0.08 | -0.44 | 0.66 |
|  | Zero | -0.14 | 0.06 | -2.31 | 0.02 |

**Processing speed**

Final model: processing speed ~ age + sex + site + smoking status + education + sleep quality + TV watching time + recreational physical activity + time-use composition

| **Variable** | **Level** | **Estimate** | **Std. Error** | **t value** | **p-value** |
| --- | --- | --- | --- | --- | --- |
| [intercept] |  | 2.89 | 0.94 | 3.10 | <0.01 |
| Age |  | -0.04 | 0.01 | -2.78 | <0.01 |
| Sex | Female | -0.04 | 0.09 | -0.41 | 0.68 |
| Site | Newcastle | -0.15 | 0.08 | -1.75 | 0.08 |
| Smoking status | Never smoked | 0.00 | 0.08 | 0.05 | 0.96 |
|  | Previous smoker | -0.13 | 0.32 | -0.40 | 0.69 |
| Education (years) |  | 0.00 | 0.01 | 0.33 | 0.75 |
| Sleep quality | “Good” | 0.06 | 0.11 | -0.55 | 0.59 |
| TV watching time | Low | 0.00 | 0.09 | 0.00 | 0.99 |
|  | Medium | 0.02 | 0.09 | 0.23 | 0.82 |
| Recreational physical activity | Under 30 minutes | -0.18 | 0.18 | -1.55 | 0.12 |
|  | Zero | -0.12 | 0.95 | -1.25 | 0.21 |
| Time-use composition | *ilr* 1 (sleep:remaining) | 0.29 | 0.26 | 1.09 | 0.28 |
|  | *ilr* 2 (SB:[LPA+MVPA]) | 0.25 | 0.16 | -1.52 | 0.13 |
|  | *ilr* 3  (LPA:MVPA) | 0.22 | 0.15 | -1.47 | 0.14 |
|  |  |  |  |  |  |

**Note:** *ilr* = isometric log-ratio; LPA = light physical activity; MVPA = moderate-vigorous physical activity. All three *ilr* coordinates must be included in the regression model for analyses, however typically only the first *ilr* is interpreted as this coordinate contains information about all four time-use behaviours (and therefore, a four-part time-use composition). An explanation of these methods can be found in the methods section of the manuscript.
